# The automation of routine light transmission platelet aggregation

**DOI:** 10.1111/ijlh.12161

**Published:** 2013-11-14

**Authors:** A S Lawrie, K Kobayashi, P J Lane, I J Mackie, S J Machin

**Affiliations:** * Haemostasis Research Unit, Department of Haematology, University College LondonLondon, UK; † Sysmex CorporationKobe, Japan; ‡ Hyphen BiomedNeuville sur Oise, France

**Keywords:** Automation, platelet aggregation

## Abstract

**Introduction:**

The investigation of platelet function by aggregometry requires specialist equipment and is labour intensive. We have developed an automated platelet aggregation method on a routine coagulation analyser.

**Methods:**

We used a CS-2000i (Sysmex) with prototype software to perform aggregation in platelet-rich plasma (PRP), using the following agonists: ADP (0.5–10 *μ*m), epinephrine (0.5–10 *μ*m), collagen (0.5–10 mg/*μ*L), ristocetin (0.75–1.25 mg/mL) and arachidonic acid (0.12–1.0 mm). Platelet agonists were from Hyphen Biomed, and an AggRAM aggregometer (Helena Biosciences) was used as the reference instrument.

**Results:**

CS-2000i reaction cuvette stirrer speed was found to influence reaction sensitivity and was optimized to 800 rpm. There were no clinically significant changes in aggregation response when the PRP platelet count was 150–480 x 10^9^/L, but below this there were changes in the maximum amplitude (MA) and slope (rate). Dose response with each of the agonists was comparable between CS-2000i and an AggRAM aggregometer and normal subjects receiving antiplatelet drugs. Aggregation imprecision was similar on both the CS-2000i and AggRAM systems, with a cv for 2–5 *μ*m ADP MA and slope varying between 3–12%.

**Conclusion:**

Our preliminary studies indicated that optimal sensitivity using the CS-2000i was obtained with a reaction cuvette stirrer speed of 800 rpm and a PRP platelet count of 200–300 x 10^9^/L; aggregation with a PRP count <100 x 10^9^/L showed poor sensitivity. Imprecision and detection of antiplatelet drug effects was similar between the CS-2000i and AggRAM. These data demonstrate that CS-2000i is comparable to a stand-alone aggregometer, although CS-2000i has the advantages of walk-away technology and also required a smaller sample volume than the AggRAM (44% less).

## Introduction

The assessment of platelet aggregation to a range of agonists including ADP, epinephrine, collagen, arachidonic acid and ristocetin [1, 2] is central to the investigation of platelet function disorders, but is only undertaken by a few specialized haemostasis laboratories [3]. Aggregation is most commonly measured by light transmission aggregometry (LTA), in which the increase in light transmission through a stirred suspension of platelet-rich plasma (PRP) is monitored as platelets aggregate. However, the manually operated instrumentation has changed little over the last 50 years [4, 5], and the process is time-consuming and labour intensive. We have previously developed an automated platelet-based ristocetin cofactor assay (VWF:RCo) on a Sysmex CS-2000i analyser (Sysmex Corporation, Kobe, Japan) [6], and in the current study, we have investigated the potential of using a similar analytical procedure on the same high-throughput coagulation analyser with prototype software to automate platelet aggregation studies.

The CS-2000i is an open analytical system, which means that test protocols and reagents can be user-defined. For this study, test protocols varying only in the reagent/concentration used were defined for commonly used platelet agonists. These protocols facilitated the generation of measured raw data (light transmission) under highly controlled conditions including sample volume, incubation period, reagent volume, reaction mixture stirrer speed and period of time for which the reaction was to be monitored. CS-2000i test protocols also contain provisions for the calculation of derived parameters, which were used to generate numeric information relating to the platelet aggregation trace from light transmission at the start of the monitoring, maximal and final transmission (which will differ when there has been disaggregation).

Performance of either platelet aggregation or coagulation tests on the CS-2000i is mutually exclusive. To request platelet aggregation studies, the analyser's *platelet mode* is selected, which makes a menu available with different platelet agonists, as opposed to *normal mode* when different coagulation tests would be displayed. The platelet aggregation software requires platelet-poor plasma (PPP) and PRP samples for each test subject, placed in consecutive positions of the analyser's sample rack. In this way, 100% light transmission is determined only once for a range of agonists, prior to assessing the PRP responses. On completion of reaction monitoring for all agonists selected for an individual's PRP, the 100% light transmission reading is integrated with the measured and derived parameters from PRP reaction monitoring. The aggregation traces are then viewed from the analyser *results file*. Operator-selected agonist traces can then be printed or stored as a portable document format (*pdf*) file in the form of an aggregation report. Raw data can also be exported to Microsoft® Excel® to facilitate the comparison of traces from different days.

## Samples and Methods

Samples were obtained with informed consent from normal healthy subjects (*n* = 14) who were not receiving any medication or who were self-medicating with nonsteroidal anti-inflammatory drugs (i.e. aspirin or ibuprofen) and from two individuals who were taking clopidogrel [7]. Blood was taken into vacutainers (BD, Oxford, UK) containing buffered sodium citrate 0.105 m (approximately 3.2%), which yields a whole-blood-to-anticoagulant ratio of 9 : 1. The sample tubes were mixed by gentle inversion before centrifugation at 170 ***g*** for 10 min at ambient temperature (approximately 20–22 °C). The resultant PRP was aspirated using plastic pipettes and transferred to polypropylene tubes (Camlab Ltd, Cambridge, UK), capped and kept at room temperature until testing. To produce PPP, the primary sample tube was centrifuged again, at 2000 ***g*** for 15 min at ambient temperature. The PPP was removed as described for the PRP. Platelet counts were then performed on the PPP and PRP using a Sysmex KX-21 cell counter (Sysmex UK Ltd). The PRP platelet count was adjusted with autologous PPP.

We assessed the ability of a CS-2000i with prototype software to perform platelet aggregation, examining the effect of varying reaction cuvette stirrer speed and the platelet count in PRP using ADP (0.5–10 *μ*m) and collagen (0.5–10 *μ*g/mL); dose response with ADP (0.5–10 *μ*m), epinephrine (0.5–10 *μ*m), collagen (0.5–10 *μ*g/mL), ristocetin (0.75–1.25 mg/mL), arachidonic acid (0.12–1.0 mm); imprecision of response to ADP (2 *μ*m and 5 *μ*m). All PRP samples were additionally monitored for 20 min without the addition of an agonist to test for spontaneous aggregation, and this ensured that results from any activated samples could be excluded from the study. To initiate reaction timing/monitoring, the CS-2000i must add a reagent to the reaction mixture. When testing for spontaneous aggregation/effect of carryover, the platelet agonist was substituted with an equal volume (20 *μ*L) of physiological saline.

All platelet agonists were from Hyphen Biomed (Neuville sur Oise, France), and an AggRAM aggregometer (Helena Biosciences Europe, Tyne and Wear, UK) was used as the reference instrument.

Tubes of PPP and PRP were placed in a rack on the CS-2000i analyser sampler unit. A combination of up to 40 user-defined reagents (platelet agonists at various concentrations) and 5 buffer systems was available. The instrument was run in micromode, meaning that for each reaction, the analyser aspirated PPP or PRP from the respective primary sample tube (140 *μ*L per test, compared to 250 *μ*L for the AggRAM) rather than taking a daughter aliquot sufficient for all requested tests. Reaction cuvettes with stirrer bars (SB-cuvettes maximum 24) were manually placed into the appropriate analyser positions using the SB-cuvette loading tool (Sysmex). On completion of reaction monitoring, CS-2000i aggregation traces were saved as*.pdf* files and raw data were exported to a Microsoft® Excel® spreadsheet.

## Results

**Figure 1:**
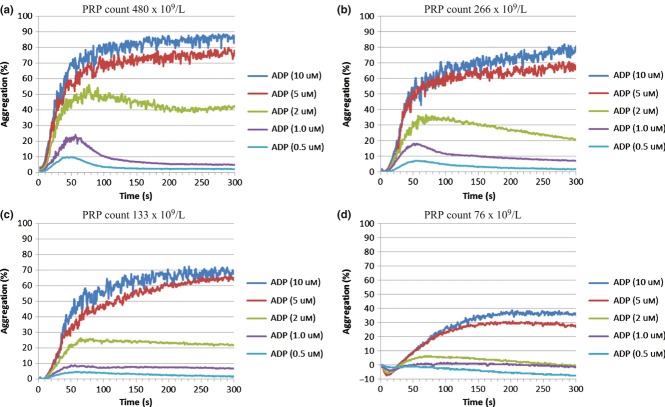
CS-2000i – Effect of platelet rich plasma platelet count on ADP dose response. The platelet rich plasma (PRP) platelet count was adjusted with autologous platelet poor plasma (PPP).

None of the PRP samples tested exhibited spontaneous aggregation on either the CS-2000i or AggRAM. There were no clinically significant changes in aggregation response when the PRP platelet count from normal healthy subjects was in the range 150–480 x 10^9^/L (Figure 1a–c), but below this there were changes in the maximum amplitude (MA) and slope (rate) of aggregation. A PRP platelet count of <100 x 10^9^/L showed poor sensitivity (Figure 1d). For further experiments, a standardized PRP count of approximately 250 x 10^9^/L was used.

**Figure 2:**
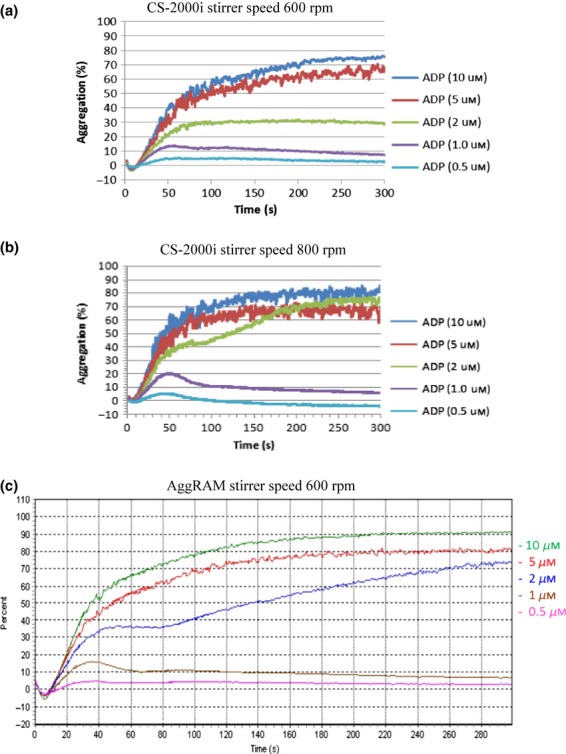
Effect of CS-2000i Stirrer speed on ADP induced aggregation.

CS-2000i reaction cuvette stirrer speed was found to influence reaction sensitivity (Figure 2a and 2b). With a stirrer speed of 800 rpm (Figure 2b), the analyser showed a similar maximal aggregation and reaction velocity to the AggRAM, which we had previously found to have an optimal stirrer speed of 600 rpm (Figure 2c). Therefore, CS-2000i cuvette stirrer speed was optimized to 800 rpm.

Aggregation imprecision for maximum amplitude and slope was similar on both analytical systems (CS-2000i: MA for ADP 2 *μ*m cv 5%, 5 *μ*m, cv 12% slope 2 *μ*m cv 6%, 5 *μ*m cv 10%. AggRAM: MA for 2 *μ*m cv 9%, ADP 5 *μ*m cv 6%, slope 2 *μ*m cv 7%, 5 *μ*m cv 3%).

**Figure 3:**
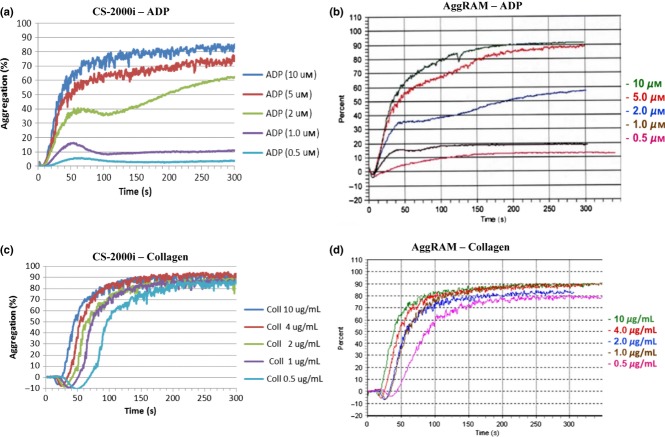
Representative dose response traces for ADP and Collagen from CS-2000i and AggRAM.

Dose response with ADP, epinephrine, collagen, ristocetin and arachidonic acid was comparable between CS-2000i and AggRAM. Representative ADP and collagen traces from CS-2000i and AggRAM are shown in Figure 3.

**Figure 4:**
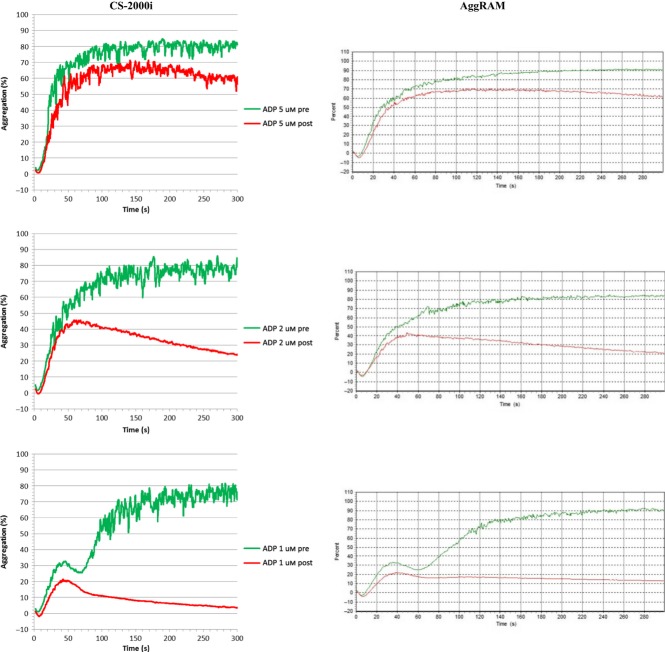
Representative ADP traces (at 1, 2 and 5 μm ADP) from CS-2000i and AggRAM Pre and 24 h Post ingestion of 75 mg aspirin.

**Figure 5:**
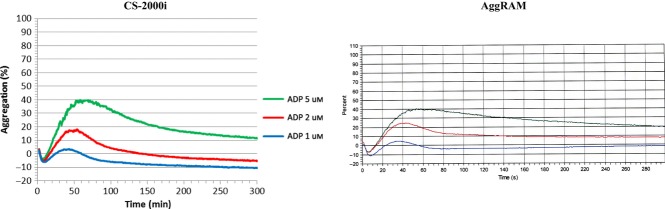
Representative ADP traces (at 1, 2 and 5 μm ADrP) from CS-2000i and AggRAM for an individual on a daily Clopidogrel maintenance dose of 75 mg.

Samples from subjects who were self-medicating with nonsteroidal anti-inflammatory drugs or taking clopidogrel exhibited comparable aggregation traces between CS-2000i and AggRAM (Figure 4). In one subject two hours after the ingestion of 400 mg ibuprofen, no aggregation was seen in response to 1.0 mm arachidonic acid, while a normal response was seen with ADP (2 *μ*m and 5 *μ*m), collagen (1 *μ*g/mL and 4 *μ*g/mL), epinephrine (2 *μ*m and 5 *μ*m) and ristocetin (1.0 mg/mL). Similarly in three subjects 24 h after taking 75 mg aspirin, complete inhibition of 1.0 mm arachidonic acid-induced aggregation was demonstrated on CS-2000i and AggRAM. The responsiveness of platelets from the aspirin group to the other agonists was variable between individuals, but similar on CS-2000i and AggRAM (Figure 4). Two individuals receiving a clopidogrel maintenance dose of 75 mg only exhibited primary aggregation with ADP (Figure 5), but normal responsiveness to the other agonists as a stand-alone diagnostic test.

## Discussion

Although platelet aggregation studies are an essential aspect of investigating platelet function disorders, a questionnaire circulated to participants of the UK National External Quality Assurance Scheme for Blood Coagulation suggested that relatively few haemostasis laboratories undertake this form of analysis [3]. However, in those laboratories that do perform platelet function testing, LTA is the method most widely used. Other tests to analyse platelet function such as whole-blood impedance aggregation [8, 9], multiplate [10–12], PFA-100 [13, 14] have not replaced LTA. Traditional aggregometers have significant capital and maintenance costs and are labour intensive. Because blood samples for platelet function testing cannot be stored, patients must be referred to specialist centres for LTA investigation, which is inconvenient to the patient and delays the diagnosis.

Perhaps because of the low number of centres performing these studies, the development of new analytical systems seems to be lagging behind the technologies used in other clinical laboratory disciplines where fully automated instrumentation is commonplace. An example of the archaic requirements of a conventional platelet aggregometer lies in the need to have each reaction channel set for zero light absorbance (i.e. blanked) with PPP prior to monitoring each individual PRP/agonist reaction. In contrast, the photo-optical system of the CS-2000i is tightly regulated, because the analyser continuously monitors the voltages from detectors (when the reaction channels are empty) to ensure that the system remains within precisely defined limits. This means that it is only necessary to take the PPP reading in one of the four SB-cuvette monitoring channels, and this single reading is applied to all reaction channels for aggregation tests associated with that sample.

Once various concentrations of platelet agonists, that is, ADP, epinephrine, collagen, arachidonic acid and ristocetin, and SB-cuvette have been loaded onto the CS-2000i, the analyser will automatically sample a patient's PPP prior to assessing that patient's PRP response to multiple agonists at a range of concentrations, without further operator interaction. Although this may not be any more rapid than performing the study manually, it does not require the constant attendance of an experienced operator. Furthermore, because the CS-2000i is an open analytical system, protocols for additional agonists could theoretically be used, such as gamma-thrombin, U-46619, collagen-related peptide, convulxin, TRAP peptides, calcium ionophore – A23187, phorbol 12-myristate 13 acetate [2], allowing the further investigation of specific patient samples.

Our preliminary studies indicated that optimal sensitivity using the CS-2000i was obtained with a reaction cuvette stirrer speed of 800 rpm and a PRP platelet count of 200–300 x 10^9^/L. Aggregation with a PRP count of <100 x 10^9^/L was detectable, but showed poor sensitivity and if performed (e.g. in thrombocytopenic patients), a normal control PRP diluted to a similar platelet count would have to be used (2). Aggregation imprecision, agonist dose response and response to platelet inhibitory medication were comparable between the CS-2000i and AggRAM. CS-2000i has the advantages of walk-away technology and also requires a smaller sample volume than the AggRAM. A panel of 5 aggregation agonists (e.g. single doses of ADP, collagen, arachidonic acid, epinephrine and ristocetin) would use approximately 0.7 mL of PRP *vs*. 1.25 mL on an AggRAM. This use of 44% less PRP would be a considerable benefit, particularly for the investigation of paediatric patients. In conclusion, we feel that these data and performance characteristics are comparable to stand-alone light transmission aggregometry and would potentially allow haemostasis laboratories to undertake a highly standardized assessment of platelet function abnormalities and responsiveness to antiplatelet drugs.

## References

[1] Cattaneo M, Cerletti C, Harrison P, Hayward CP, Kenny D, Nugent D, Nurden P, Rao AK, Schmaier AH, Watson SP, Lussana F, Pugliano MT, Michelson AD (2013). Recommendations for the standardization of light transmission aggregometry: a consensus of the working party from the platelet physiology subcommittee of SSC/ISTH. J Thromb Haemost.

[2] Harrison P, Mackie I, Mumford A, Briggs C, Liesner R, Winter M, Machin S (2011). Guidelines for the laboratory investigation of heritable disorders of platelet function. Br J Haematol.

[3] Jennings I, Woods TA, Kitchen S, Walker ID (2008). Platelet function testing: practice among UK National External Quality Assessment Scheme for Blood Coagulation participants, 2006. J Clin Pathol.

[4] Born GV, Cross MJ (1963). The aggregation of blood platelets. J Physiol.

[5] O'Brien JR (1962). Platelet aggregation: part II Some results from a new method of study. J Clin Pathol.

[6] Lawrie AS, Mackie IJ, Machin SJ, Peyvandi F (2011). Evaluation of an automated platelet-based assay of ristocetin cofactor activity. Haemophilia.

[7] Izaguirre-Avila R, Pena-Diaz A, Barinagarrementeria-Aldatz F, Gonzalez-Pacheco H, Ramirez-Gutierrez AE, Ruiz-Sandoval JL, Quiroz-Martinez A, Cantu-Brito C (2002). Effect of clopidogrel on platelet aggregation and plasma concentration of fibrinogen in subjects with cerebral or coronary atherosclerotic disease. Clin Appl Thromb Hemost.

[8] Dyszkiewicz-Korpanty AM, Frenkel EP, Sarode R (2005). Approach to the assessment of platelet function: comparison between optical-based platelet-rich plasma and impedance-based whole blood platelet aggregation methods. Clin Appl Thromb Hemost.

[9] Dyszkiewicz-Korpanty AM, Kim A, Burner JD, Frenkel EP, Sarode R (2007). Comparison of a rapid platelet function assay–Verify Now Aspirin–with whole blood impedance aggregometry for the detection of aspirin resistance. Thromb Res.

[10] Lee KR, Verheyden VJ, Mumford AD (2012). Evaluation of multiple electrode aggregometry in whole blood using Multiplate Mini Test cells. Thromb Res.

[11] Valarche V, Desconclois C, Boutekedjiret T, Dreyfus M, Proulle V (2011). Multiplate whole blood impedance aggregometry: a new tool for von Willebrand disease. J Thromb Haemost.

[12] Pedersen SB, Grove EL, Nielsen HL, Mortensen J, Kristensen SD, Hvas AM (2009). Evaluation of aspirin response by Multiplate whole blood aggregometry and light transmission aggregometry. Platelets.

[13] Harrison P (2004). In vitro measurement of high-shear platelet adhesion and aggregation by the PFA-100. Methods Mol Biol.

[14] Harrison P (2005). The role of PFA-100 testing in the investigation and management of haemostatic defects in children and adults. Br J Haematol.

